# Acute Respiratory Infections

**Published:** 1918-10

**Authors:** 


### THE WINTER CAMPAIGN AGAINST ACUTE RESPIRATORY INFECTIONS.

The season is approaching when the infections of the respiratory tract must be regarded as enemies more dangerous to our Armies than the Prussian soldiers fighting under Hindenburg; and it is comforting to knowof the thorough preparations that are being made to combat the two types of parasites that are a menace to American soldiers in France, i. e., germs and Germans. The Chief Surgeon of the American Expeditionary Forces, aided by a staff of expert sanitarians and clinicians, is leading the fight on the streptococcus, pneumococcus, and meningococcus — enemies that killed so many good American soldiers last winter; while Marshal Foch and General Pershing are seeing to it that the poisonous gases, and the fire of shot and shell that are thrown out by the parasites of the genus hominis Germani group in their efforts to overcome their unwilling hosts, are met with a resistance that will destroy the invaders, or drive them back into Kaiserland — the only soil suitable for their growth and development, and where they must remain in ignominious retirement for ever.

As a part of the winter campaign against acute respiratory infections, one session of the September meeting of the Research Society was devoted to the discussion of that subject. Medical officers representing various medical units, both from the field and hospitals, had the opportunity at this meeting to hear the experience of the British and French with diseases of the respiratory tract, and they were also informed of the plans of the Medical Department of the American Expeditionary Forces for the winter campaign against this group of diseases.

### Experience of the British and French

The British Armies in France have had remarkably good results in preventing respiratory diseases. Their statistics show much lower death and non-effective rates from this group of infections than has been the experience of the American troops in the United States and France. Colonel Beveridge of the British Medical Service opened the symposium on respiratory infections by a description of the methods that have been used to prevent them in the British Armies. Apparently the one most effective measure has been the proper ventilation of soldiers' quarters, particulary their sleeping apartments. British regulations require an ample allowance of air space for their soldiers before they move into the houses or tents provided for them; except, of course, in a drive where troops in large numbers have to be moved rapidly, when there may be temporary overcrowding. Colonel Beveridge said that the British have also been careful to isolate their cases of the acute respiratory infections as early as possible. He thought that probably one of the reasons why the British troops had had such a low rate from the acute respiratory infections was that they were seasoned and had been taught how to take care of themselves.

Professor Borel gave an interesting account of an epidemic of pneunomia among the Senegalese troops with the French Army. He said that the native French troops have suffered comparatively little with respiratory infections; but that those from their colonial possessions showed a high rate, particularly from pneumonia. The Senegalese apparently have an inherent susceptibility to pneumonia. The death rate from this disease has been high among them, both in their own country and during their first winter in France. In this epidemic, the contagiousness, or infectiousness, of pneumonia was proved. The cases were thoroughly studied clinically, and bacteriological examinations showed that the pneumococcus of "strain No 24 of Lister's African group" was the infective agent. The serum obtained from this strain does not agglutinate with any of the American or European groups of pneumococcus. Professor Borel's report of the apparent immunity obtained by employing the pneumococcus vaccines made from the African strain found in this epidemic is suggestive and encouraging.

### Respiratory Infections in the American Armies

Major Haven Emerson said that, in the first year of the war, respiratory infections were responsible for a large proportion of the deaths among American soldiers. He thought that the great prevalence and high death rate from measles, the pneumonias, and meningitis among the American troops might be explained partly by the fact that a large proportion of our troops come from sparsely settled country districts where they have not been exposed to many infections, and they therefore have a high degree of susceptibility to them. He said that it might be that the British and French troops, living as they do in cities, and in thickly populated countries, have acquired more or less immunity to respiratory infections. He said that reports from camps in the United States indicate that intensive exhaustive drilling has been responsible for the increased susceptibility to respiratory infections among certain troops.

Major Emerson said that the colored soldiers in the United States Army had suffered more, and that they showed higher death rates from respiratory infections than the white troops; even more than in civil life, though the incidence and death rates from pneumonia among all classes of the colored population in the United States is three times that of the whites. There is apparently a racial susceptibility among the blacks to respiratory infections.

Major Emerson showed that, incident to getting together a large Army in a short time, there has been overcrowding in the quarters for troops. He hoped, however, that this could be remedied before winter. It is evident, he said, that there is a direct relationship to the amount of air-space provided for soldiers and the prevalence of acute respiratory infections.

Major Emerson further spoke of the need for an immediate campaign of education to teach our troops the dangers from coughing and spitting, and of the necessity for proper ventilation of their working and living quarters. He thought that the question of acute respiratory infections is one of housekeeping, environment, discipline, and sanitation.

THE SUSCEPTIBILITY, EXHAUSTION, AND CONTACT FACTORS

Major Zinsser, who had investigated some of the epidemics of pneumonia in American camps, thinks that in pneumococcus infections contact is not so important as the hygienic, or susceptibility, problem. He said that in the same tent he has found types 1, 2, and 4 of the pneumococcus, though in a number of instances the strains were of the same type, indicating contact infection.

As to the causes of the epidemics in the American Camps, he mentioned overcrowding as important, though the " overwork factor " played a prominent part. There was a very distinct improvement in conditions when the drill was not exhaustive. The method of gradually introducing soldiers to army life is to be recommended as a most excellent system in preventing respiratory infections. He said that the death rate from primary pneumonia was 12 o/o, and that the secondary type following measles was 29 o/o. It is self-evident that the prevention of measles is important, and that when measles appears in a camp prompt isolation of those infected, and daily or twice-daily inspections of all troops in the unit, should be made. He said that contact infection was the most important etiological factor in the secondary pneumonias due to the streptococcus.

Major Zinsser said that meningitis is in the same category as lobar pneumonia; that it is a question of susceptibility more than contact infection. He was not sure that the carrier had much to do with the prevalence of meningitis. He thought the best methods of prevention are "rate determination" and careful bacteriological study of the men who have come in contact with an infected individual.

General Burtchaell said that air space in itself is not nearly so important as free ventilation in preventing acute respiratory infections. He thought it often better for soldiers to occupy limited air space for one or two nights and give them shelter, than to expose them to the elements outside.

### The Winter Campaign.

The symposium on acute respiratory infections was but the firing of the first gun of the winter campaign against influenza, bronchitis, measles, the pneumonias, diphtheria and meningitis and other winter diseases.

At the October meeting of the Research Society the weak points in the defence of General Streptococcus and his death-dealing hordes will be laid bare, and a plan of campaign will be outlined to combat this enemy of the American Army.

Other preparations on a large scale are being carried on for a big offensive against the acute respiratory infections that will begin in October and last until spring. The Chief Surgeon and the Commander-in-Chief of the American Expeditionary Forces know the wiles and the strength of the enemies of the armies which they lead; and they have the men and the resources with which to carry on the fight against them. It therefore is safe to predict that the germs and the Germans that touch the American lines, will spend an uneasy, unprofitable and altogether miserable winter in 1918-1919.

